# Risk of bacteremia in patients presenting with shaking chills and vomiting - a prospective cohort study

**DOI:** 10.1017/S0950268820000746

**Published:** 2020-03-31

**Authors:** M. Holmqvist, M. Inghammar, L. I. Påhlman, J. Boyd, P. Åkesson, A. Linder, F. Kahn

**Affiliations:** 1 Division of Infection Medicine, Department of Clinical Sciences, Lund University, Lund, Sweden; 2 Department of Infectious Diseases, Skåne University Hospital, Lund, Sweden; 3 Centre for Heart Lung Innovation, University of British Columbia, Vancouver, BC, Canada

**Keywords:** Bacterial infections, bloodstream infections, chills, emergency department, septicaemia

## Abstract

Chills and vomiting have traditionally been associated with severe bacterial infections and bacteremia. However, few modern studies have in a prospective way evaluated the association of these signs with bacteremia, which is the aim of this prospective, multicenter study. Patients presenting to the emergency department with at least one affected vital sign (increased respiratory rate, increased heart rate, altered mental status, decreased blood pressure or decreased oxygen saturation) were included. A total of 479 patients were prospectively enrolled. Blood cultures were obtained from 197 patients. Of the 32 patients with a positive blood culture 11 patients (34%) had experienced shaking chills compared with 23 (14%) of the 165 patients with a negative blood culture, *P* = 0.009. A logistic regression was fitted to show the estimated odds ratio (OR) for a positive blood culture according to shaking chills. In a univariate model shaking chills had an OR of 3.23 (95% CI 1.35–7.52) and in a multivariate model the OR was 5.9 (95% CI 2.05–17.17) for those without prior antibiotics adjusted for age, sex, and prior antibiotics. The presence of vomiting was also addressed, but neither a univariate nor a multivariate logistic regression showed any association between vomiting and bacteremia. In conclusion, among patients at the emergency department with at least one affected vital sign, shaking chills but not vomiting were associated with bacteremia.

## Introduction

Fever and chills are common symptoms in the emergency department. Fever is generally considered a symptom of infection, but also occurs frequently in other inflammatory diseases and trauma [1]. Chills are caused by muscles rapidly contracting and relaxing in response to a raised body temperature balance point. Pyrogenic cytokines and microbial products can also cause systemic symptoms such as vomiting [1]. Chills have traditionally been associated with bacteremia [2], however few modern prospective studies exist [3–9].

Bacteremia is defined as the presence of bacteria in blood, which can be part of a severe infection. The subsequent immune response may lead to the development of the sepsis syndrome. Rapid recognition and initiation of antibiotics are of great importance for survival [10], therefore it is important to rapidly identify patients with bacteremia or a severe bacterial infection. Conversely, it is important at the population level to identify patients who do not have a bacterial infection to avoid unnecessary use of antibiotics. Other investigators have developed clinical prediction rules for the risk of bacteremia in patients with suspected infection [3, 4, 11–23]. However, these studies have yielded conflicting results. Chills and vomiting are two clinical symptoms that are widely considered cardinal symptoms of severe bacterial infections or bloodstream infections, however, this association and the combination of these symptoms have rarely been systematically studied.

The objective of our study was to determine if the presence of shaking chills and/or vomiting in the Emergency Department could predict bacteremia.

## Methods

### Study design and data collection

This is a sub analysis of the HERO study (ClinicalTrials.gov NCT 02366650) [24]. The HERO study was a prospective, multicenter, observational convenience sample cohort study. Details of the study are described elsewhere [24]. In summary, patients were enrolled upon presentation to the Emergency Department when fulfilling the following inclusion criteria: (1) age ≥18 years and (2) at least one of the following criteria irrespectively of cause of the condition: (a) respiratory rate >25 breaths per minute; (b) heart rate >120 beats per minute; (c) altered mental status; (d) systolic blood pressure <100 mm Hg; (e) oxygen saturation <90% without oxygen; (f) oxygen saturation <93% with oxygen; and (g) reported oxygen saturation <90%. The study was conducted at four university or university-affiliated hospitals during 2015 and 2016: Skåne University Hospital in Lund, Sweden; Inselspital University Hospital in Bern, Switzerland; St Paul's Hospital in Vancouver, Canada and Helsingborg Hospital in Helsingborg, Sweden. The hospitals cover areas of between 200.000 and 1.500.000 individuals and have between 45.000 and 115.000 annual visits to their respective Emergency Departments. Patients from all sites except Helsingborg were included in the present study, since no structured data collection regarding shaking chills was carried out in Helsingborg.

The collected clinical characteristics include demographics, comorbidities, current medication, information about the current disease (including precise information about the presence/history of shaking chills and vomiting), vital signs, laboratory testing, given treatment, organ dysfunction (OD), intensive care, mortality, and final diagnosis.

Blood cultures were obtained on request of the treating clinician at the Emergency Department, independent of study inclusion. Automated systems for blood cultures were used; BACTEC FX (Becton Dickinson) in Lund and Vancouver and BacT-Alert (Biomeriux) in Bern. Blood was drawn by nurses and two sets of bottles were obtained (one set consisting of one aerobic and one anaerobic bottle, each bottle containing 10 mL of blood). In exceptional cases with procedural problems only one set of bottles or a smaller blood volume would be obtained. Blood was normally incubated for 5 days, in the case of prolonged incubation it was incubated for 14 days in Lund and Vancouver, and for 10–21 days in Bern.

### Definitions

Shaking chills were defined as involuntarily shivering such that holding a glass of water in the hand would cause the water to spill out. Fever was defined as body temperature ≥38.0 °C. Vomiting was defined as vomiting within the last 24 h preceding the Emergency Department visit. Patients were asked by the study team at inclusion for the presence or history of shaking chills or vomiting. Positive blood cultures were defined as the growth of a microorganism with known pathogenicity in at least one blood culture or the growth of a microorganism commonly known as a skin pathogen (e.g. Coagulase-negative *Staphylococcus* or *Cutibacterium acnes*) in at least two blood cultures, together with a clinical assessment of a related infection.

The presence of an infection was assessed retrospectively by two independent Infectious Diseases consultants, with the addition of a third Infectious Diseases consultant if needed to reach consensus, and graded in six separate categories: (i) Verified bacterial infection was defined as a microbiological and/or radiological finding together with symptoms of infection from the same site, or a positive blood culture with a significant bacterial pathogen. (ii) Probable bacterial infection was considered an infection by the attending physician, but not fulfilling the criteria for a verified bacterial infection. (iii) Viral infection was defined as the microbiological finding of a viral pathogen in concordance with the present symptoms and the absence of a bacterial cause. (iv) Probable viral infection was defined as a viral infection diagnosed by the attending physician with no use of antibiotics and with laboratory and microbiological findings in agreement. (v) Probably no infection was defined as a condition not considered a bacterial or viral infection according to the attending physician but not fulfilling the criteria for a verified non-infection. (vi) Verified non-infection was defined as a non-bacterial and non-viral condition, without positive cultures or the use of antibiotics, and with survival at least 12 h after inclusion.

For the assessment of OD among infected patients the criteria for OD was used as previously described [24]. The definition of OD was adapted from the sepsis-2 consensus criteria although the fulfillment of two SIRS-criteria was not necessary due to the lack of validity [25, 26]. We used the sepsis-2 definition since this was the definition in use when the data was collected. Briefly, OD was defined as present when any definition of OD was met within 72 h in the absence of preexisting pathology that could explain the abnormal results. Plasma lactate was not included as a criterion for OD.

### Ethical considerations

The regional ethical boards approved the trial at each center (Lund 2014/41, Bern KEK 315/14, Vancouver H11-00505).

### Statistical analysis

Means, medians, standard deviations (s.d.s), and interquartile ranges (IQRs) were reported when appropriate. Differences in frequencies between groups were tested with Fisher's exact test and differences between group medians with the Mann–Whitney *U* test. The odds ratios (ORs) for bacteremia were calculated according to the presence of shaking chills or vomiting using separate logistic regression models. Sex and age were *a priori* considered potential confounders to the association to bacteremia and were included in a multivariate model. Temperature was not included as a covariate since it was deemed to be on the same biological pathway as chills. However, to investigate if there was an interaction between shaking chills and temperature, temperature as well as the interaction between temperature and shaking chills were included in such an analysis. For three patients there were no available information about temperature and hence they were excluded from this latter analysis. To account for possible dependency due to sampling at four different sites, we used mixed model with random effects and generalized estimating equation (GEE) models with site as the random components.

The statistical package R (Vienna, Austria. URL https://www.R-project.org/) was used for statistical computation with the following modules: readxl, haven, dplyr, epiR, MASS and, Hmisc, ggplot2, geepack, lme4 and MuMln. *P*-Values lower than 0.05 were regarded as significant. Likelihood ratio tests were used to test for interactions.

## Results

**Fig. 1.**
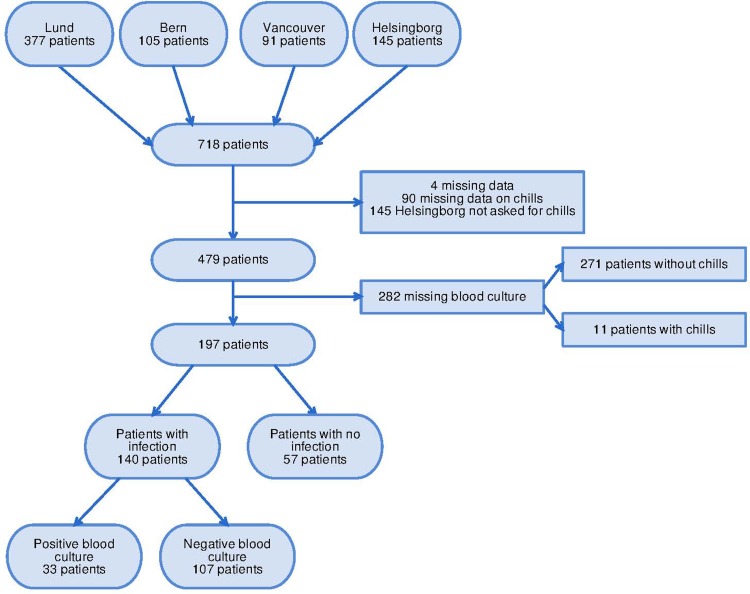
Flow chart of patients in the study cohort.

In total 718 patients were included in the HERO study protocol (377 from Lund, 105 from Bern, 91 from Vancouver and 145 from Helsingborg). Ninety-six patients were excluded because of missing data on chills, four patients with missing diagnoses and the 145 patients from Helsingborg were excluded since they were not structurally asked for the presence of shaking chills (Fig. 1). This yielded 479 patients. The demographics are showed in Table 1.

**Table 1.** Baseline characteristics

	*N*	No chills *N* = 434	Chills *N* = 45	*P*-Value
**Site**	479			0.4
**Lund**		65% (283)	58% (26)	
**Bern**		21% (91)	22% (10)	
**Vancouver**		14% (60)	20% (9)	
**Type of problem**	479			7 × 10^−6^
**Verified bacterial infection**		15% (64)	33% (15)	
**Probable bacterial infection**		15 % (63)	18% (8)	
**Verified viral infection**		6% (27)	16% (7)	
**Probable viral infection**		1% (5)	7% (3)	
**Probable no infection**		12% (51)	9% (4)	
**No infection**		52% (224)	18% (8)	
**Prior antibiotics**	477	9% (41)	22% (10)	0.02
**Vomiting**	478	15% (64)	32% (14)	0.008
**Fever**	463	17% (69)	60% (27)	1 × 10^−9^
**Age**	479	70.5 (56.8–80.7)	65.9 (53.5–79.9)	0.3
**Females**	479	50% (217)	47% (21)	0.8
**Comorbidities**				
**Cardiovascular**	479	50% (219)	44% (20)	0.5
**Respiratory**	479	24% (105)	27% (12)	0.7
**Diabetes**	479	20% (85)	20% (9)	1
**Renal**	479	15% (65)	20% (9)	0.4
**Malignancy**	479	13% (58)	16% (7)	0.6
**COPD**	479	12% (50)	13% (6)	0.6
**Liver**	479	3% (15)	11% (5)	0.03
**Immunodeficiency**	479	2% (9)	7% (3)	0.09
**No comorbidities**	479	27% (119)	29% (13)	0.9
**Outcome**				
**ICU-admittance within 72 h**	478	10% (42)	7% (3)	0.8
**Mortality within 72 h**	479	3% (12)	2% (1)	1
**Number of dysfunctional organs**	479			0.6
**0**		38% (167)	38% (17)	
**1**		29% (126)	33% (15)	
**2**		18% (80)	13% (6)	
**3**		6% (26)	9% (4)	
**4**		4% (16)	2% (1)	
**5**		1% (6)	0% (0)	
**6**		0% (1)	2% (1)	
**7**		3% (12)	2% (1)	
**Diagnoses**	479			0.004
**Infections**		37% (162)	73% (33)	
**Respiratory**		15% (63)	16% (7)	
**Influenza**		4% (18)	13% (6)	
**Genitourinary**		4% (17)	13% (6)	
**COPD exacerbation**		3% (13)	2% (1)	
**Unspecified sepsis**		3% (12)	4% (2)	
**Gastrointestinal**		3% (11)	7% (3)	
**Other bacterial infection**		3% (11)	2% (1)	
**Other viral infections**		2% (10)	7% (3)	
**Skin/soft tissue**		1% (5)	4% (2)	
**Endocarditis**		0% (2)	4% (2)	
**Non-infectious causes**		63% (272)	27% (12)	
**Other**		14% (60)	9% (4)	
**Heart rhythm**		11% (48)	0% (0)	
**Gastro**		8% (33)	2% (1)	
**Lung**		7% (30)	7% (3)	
**Unspecified heart**		7% (29)	2% (1)	
**Orthopedic**		3% (13)	0% (0)	
**Cerebrovascular**		3% (12)	2% (1)	
**Lung embolic**		2% (10)	0% (0)	
**Intoxication**		2% (8)	0% (0)	
**CNS**		1% (6)	0% (0)	
**Seizures**		1% (6)	0% (0)	
**Kidney**		1% (4)	4% (2)	
**Diabetes**		1% (4)	0% (0)	
**Vascular**		1% (3)	0% (0)	
**Head trauma**		0% (2)	0% (0)	
**Acute myocardial infarction**		0% (2)	0% (0)	
**Liver**		0% (1)	0% (0)	
**Psychiatric**		0% (1)	0% (0)	

Baseline characteristics of the total population (*n* = 479). Continuous variables are displayed with a median and inter-quartile range. Categorical variables are displayed with proportions and numbers within brackets. Non-categorical variables are tested with the Mann–Whitney *U* test and categorical variables with Fisher's exact test.

### Shaking chills and bacterial infection

The association of shaking chills with bacterial infection was analyzed. Forty-five patients (9%) reported the presence of shaking chills. One hundred and fifty patients (31%) had a verified or probable bacterial infection. Of these, 23 patients (15%) reported the presence of shaking chills compared to 22 (7%) of the 329 patients without a bacterial infection, *P* < 4 × 10^−6^. The presence of shaking chills according to type of infection is displayed in Table 1.

### Shaking chills and bacteremia

Out of the 479 included patients, 197 patients had blood cultures performed and were thus eligible for inclusion in the analysis of the association of shaking chills with bacteremia. The demographics of these patients are showed in supplementary Table S1. Of these, 32 patients (16%) had a positive blood culture. Thirty-four patients (17%) reported the presence of shaking chills. Of the 32 patients with a positive blood culture 11 patients (34%) had experienced shaking chills compared with 23 (14%) of the 165 patients with a negative blood culture, *P* = 0.009.

A logistic regression was fitted to show the estimated OR for a positive blood culture according to shaking chills both with and without covariates. Results are displayed in Table 2. Model 1 is a univariate model which shows an OR = 3.23 (95% CI 1.35–7.52) for a positive blood culture according to shaking chills. To adjust for confounding factors a multivariate model (model 2) was fitted adjusting for sex and age (65–80 years and >80 years, respectively), yielding an OR = 3.72 (95% CI 1.51–9.06) for a positive blood culture according to shaking chills. Fever was not included as a covariate since it was deemed to be on the same biological pathway as chills. However, to assess a potential interaction between fever and shaking chills, fever (≥38.0 °C) was included both as a covariate and as an interaction term with shaking chills (model 2b). Neither fever nor the interaction of fever with shaking chills was significant (*P* = 0.67 and *P* = 0.64). Models 2 and 2b were also compared with a likelihood test and model 2b was not significantly better than model 2 (*P* = 0.66). Hence, there was no statistical support for an effect modification by fever.

**Table 2.** Association between shaking chills and bacteremia

		Age				
Model	Chills	65–80 years	>80 years	Sex	Antibiotics prior	Chills: Antibiotics prior
1	3.23 (1.35–7.52) *P* = 0.007	–	–	–	–	–
2	3.72 (1.51–9.06) *P* = 0.004	2.64 (0.95–8.18) *P* = 0.07	4.00 (1.41–12.66) *P* = 0.01	0.84 (0.37–1.85) *P* = 0.66	–	–
3	5.89 (2.05–17.17) *P* = 0.0003	2.56 (0.90–8.13) *P* = 0.09	5.01 (1.67–17.0) *P* = 0.006	0.81 (0.35–1.84) *P* = 0.62	3.29 (1.01–10.11) *P* = 0.04	0.12 (0.01–1.14) *P* = 0.07

OR (95 % CI). Univariate and multivariate logistic regression models with estimated ORs for a positive blood culture according to shaking chills with and without adjustment for age, sex, and prior antibiotics among patients where blood cultures have been obtained (*n* = 197).

Of the 197 patients in the cohort, 31 patients were treated with antibiotics prior to the ED encounter. Since antibiotics prior to blood culture can affect the yield of the blood culture a model to account for this was constructed. One patient was excluded since information about prior antibiotics was missing. In this model prior antibiotics as well as the interaction between shaking chills and prior antibiotics was added (model 3). In this model shaking chills had an OR of 5.89 (95% CI 2.05–17.17) among those *without* prior antibiotics and an OR of 0.72 (95% CI 0.08–4.86) among those *with* prior antibiotics. However, the difference in OR among the subgroups was not significant (*P* = 0.07). As a sensitivity analysis, patients with prior antibiotics were excluded and a logistic regression with shaking chills, age, and sex as covariates was fitted. In this model shaking chills had an OR = 6.68 (2.24–21.04).

### Influence of the seasonal incidence of viral infections

**Fig. 2.**
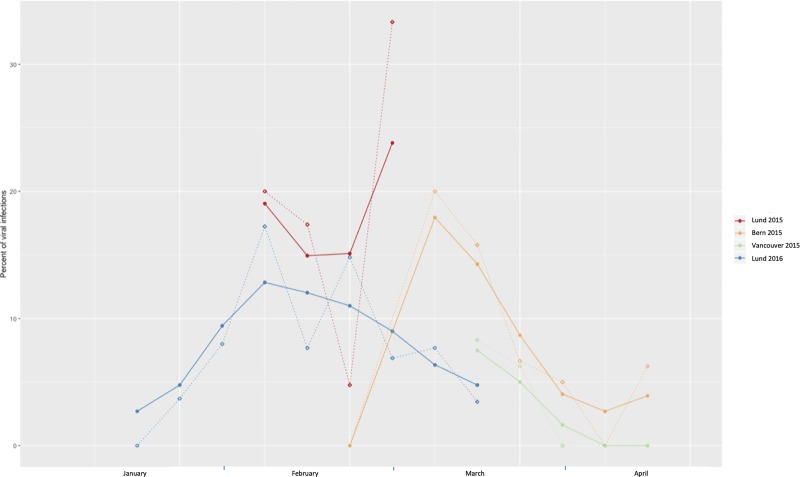
Weekly incidence of viral infections per site and inclusion year. Only weeks with a minimum of two patients with infections are included in the figure. Dashed line represent the exact incidence, solid line represents the smoothed incidence.

The patients were included at all the three different sites during January to March 2015 and again in Lund during January to March 2016. The weekly incidence of viral infections among included patients (*n* = 479) per site was assessed. For some weeks the number of included patients at a given site was low and therefore a smoothed incidence was constructed. This smoothed incidence was the mean of the incidence for the preceding, current, and following week at the specific site but with the incidence for the current week weighted by a factor of 2 (Fig. 2). This shows a maximal weekly incidence of viral infections among included patients during 2015 of 33% in Lund, 8% in Vancouver, 20% in Bern, and 17% in Lund during 2016. As shown in Table 1, 24% of those with viral infections did experience shaking chills. Since the inclusion in the study took place during the influenza season, the incidence of viral infections might have affected the prognostic properties of shaking chills. To account for this, a new logistic regression was fitted in the population where blood cultures had been obtained (*n* = 197). For each site the weighted weekly incidence of viral infections was added in a logistic regression together with age, sex, and prior antibiotics (model 4, Table 3). The interactions between shaking chills and the incidence of viral infections, as well as the interaction between shaking chills and prior antibiotics use were also evaluated in the same model. The result shows an OR of 31 (95% CI 4.60–280.82) for a positive blood culture according to shaking chills when adjusted for viral infections. However, there was a considerable interaction, although not significant, between viral incidence and chills (OR 0.82, 95% CI 0.64–1.00).

**Table 3.** Association between shaking chills and bacteremia additionally adjusted for viral incidence

Model	Chills	Age		Sex	Antibiotics prior	Chills: Antibiotics prior	Viral incidence per week	Chills: Viral incidence
		65–80 years	>80 years					per week
4	31.05 (4.60–280.82) *P* = 0.0009	6.94 (1.91–34.37) *P* = 0.007	14.91 (3.78–80.54) *P* = 0.0004	0.82 (0.34–1.94) *P* = 0.66	3.55 (1.03–11.68) *P* = 0.04	0.04 (0.00–0.49) *P* = 0.02	0.91 (0.82–0.99) *P* = 0.03	0.82 (0.64–1.00) *P* = 0.08
5a	23.17 *P* = 0.0003	6.35 *P* = 0.0003	15.07 *P* = 9 × 10^−10^	0.86 *P* = 0.82	3.06 *P* = 0.003	0.07 *P* = 0.006	0.91 *P* = 2 × 10^−10^	0.84 *P* = 0.0002
5b	27.2 *P* = 0.002	6.85 *P* = 0.009	19.3 *P* = 0.0003	0.84 *P* = 0.71	2.98 *P* = 0.09	0.07 *P* = 0.05	0.91 *P* = 0.07	0.83 *P* = 0.09

OR (95 % CI). Model 4 shows a multivariate logistic regression model with estimated OR for a positive blood culture according to chills with adjustment for age, sex, prior antibiotics and viral incidence per week among patients where blood cultures have been obtained (*n* = 197). Model 5a shows a GEE with the same covariates as model 4 and model 5b shows the same model fitted using the sites as random effects and a random intercept model.

### Analysis of the effect of different sites

Finally, to account for the possible effects of the different sites, a GEE was fitted with the same covariates as model 4 (model 5a, Table 3). The same model was also fitted using the sites as random effects and a random intercept model was fitted (model 5b). The results show an OR of 23 and 27, respectively.

### Shaking chills and organ dysfunction

In a secondary analysis, we assessed the association between shaking chills and infection-induced OD. Among the 479 patients in the cohort 192 patients were adjudicated to have an infection. Of these 192 patients, 33 experienced shaking chills and 137 had or within 72 h developed OD. A logistic regression was fitted to show the estimated OR for OD according to shaking chills. Shaking chills had a crude OR = 1.31 (95% CI 0.57–3.30) and an OR = 1.45 (95% CI 0.61–3.78) when adjusted for age and sex, in predicting the presence or development of OD among infected patients.

### Vomiting and bacteremia

Vomiting is classically considered a sign of serious infection. In the cohort where blood cultures were obtained, 196 out of the 197 patients also had registered data about vomiting. Of these, 38 patients (19%) experienced vomiting during the last 24 h and five (13%) of these patients had a positive blood culture. Of the 158 patients who did not experience vomiting, 27 patients (17%) had a positive blood culture. Of the 32 patients with a positive blood culture five patients (16%) had vomited and of the 164 patients with negative blood cultures 33 patients (20%) had vomited (*P* = 0.63). The same analysis was also made among patients with no prior antibiotics: Of the 24 patients with a positive blood culture three patients (13%) had vomited and of the 140 patients with a negative blood culture 28 patients (20%) had vomited (*P* = 0.57).

A logistic regression showing the estimated OR for a positive blood culture according to vomiting both with (model 7) and without (model 6) age, sex, prior antibiotics and the interaction between vomiting and prior antibiotics as covariates are displayed in Table 4. In none of the models vomiting was significantly associated with a positive blood culture.

**Table 4.** Association between vomiting and bacteremia

Model	Vomiting	Age				
		65–80 years	>80 years	Sex	Antibiotics prior	Vomiting: Antibiotics prior
6	0.74 (0.24–1.91) *P* = 0.56	–	–	–	–	–
7	0.61 (0.13–1.98) *P* = 0.45	2.19 (0.80–6.65) *P* = 0.14	3.40 (1.22–10.46) *P* = 0.02	0.94 (0.42–2.08) *P* = 0.88	1.90 (0.61–5.34) *P* = 0.24	1.52 (0.13–16.11) *P* = 0.73

OR (95 % CI). Uni- and multivariate logistic regression models with estimated ORs for a positive blood culture according to vomiting with and without adjustment for age, sex, and prior antibiotics among patients where blood cultures have been obtained (*n* = 196).

### Combination of vomiting and shaking chills

To address the question if the combination of vomiting and shaking chills predicted bacteremia, the impact of vomiting was investigated in the subpopulation of patients with shaking chills and where blood cultures and information of vomiting were obtained. Of these 33 patients, 11 patients had a positive blood culture and out of these, three patients (27%) had vomited whereas among the 22 patients with a negative blood culture seven patients (32%) had vomited (*P* = 1). As a sensitivity analysis this analysis was repeated among the patients with no prior antibiotics. Of these 26 patients, 9 patients had a positive blood culture and out of these two patients (22%) had vomited whereas among the 17 patients with a negative blood culture six patients (35%) had vomited (*P* = 0.67). Thus, the presence of vomiting had no additional value as to the presence of positive blood cultures among patients with shaking chills.

### Shaking chills and bacterial species

The relationship between shaking chills and bacterial species was also investigated and the results are shown in Table 5. For this analysis four patients with blood cultures containing more than one species were excluded and hence 28 patients were analyzed. There was no significant association between recovered species and the presence of shaking chills (*P* = 0.68).

**Table 5.** Bacterial pathogens isolated from blood cultures

Bacterial pathogen	Total number of patients	Number of patients with chills	Proportion of patients with chills (%)
*Staphylococcus aureus*	8	2	25
*Streptococcus pyogenes*	1	1	100
*Streptococcus pneumoniae*	1	1	100
*Streptococcus mitis*	1	0	0
*Enterococcus faecalis*	1	0	0
*Enterococcus faecium*	1	0	0
*Escherichia coli*	11	4	36
*Proteus mirabilis*	1	1	100
*Pseudomonas aeruginosa*	1	0	0
*Haemophilus influenzae*	1	0	0
*Morganella morganii*	1	0	0

Bacterial pathogens isolated from blood cultures and number and proportion of patients with shaking chills.

### Properties of shaking chills or vomiting in predicting bacteremia

Next, the predictive properties of shaking chills and vomiting in predicting bacteremia were investigated and are displayed in Table 6. The presence of shaking chills had a positive predictive value of 36% whereas the presence of vomiting had a positive predictive value of 17%.

**Table 6.** Predictive properties for bacteremia of shaking chills and vomiting

	Sensitivity	Specificity	PPV	NPV	LR+	LR−
Chills	0.34 (0.19–0.53)	0.86 (0.80–0.91)	0.32 (0.17–0.51)	0.87 (0.81–0.92)	2.47 (1.34–4.54)	0.76 (0.59–0.99)
Vomiting	0.16 (0.05–0.33)	0.80 (0.73–0.86)	0.13 (0.04–0.28)	0.83 (0.76–0.88)	0.78 (0.33–1.84)	1.06 (0.89–1.25)
Chills^a^	0.38 (0.19–0.59)	0.87 (0.81–0.92)	0.33 (0.17–0.54)	0.89 (0.83–0.94)	2.94 (1.50–5.76)	0.72 (0.52–0.98)
Vomiting^a^	0.12 (0.03–0.32)	0.80 (0.72–0.86)	0.10 (0.02–0.26)	0.84 (0.77–0.90)	0.63 (0.21–1.89)	1.09 (0.92–1.30)

PPV, positive predictive value; NPV, negative predictive value; LR+, positive likelihood ratio; LR−, negative likelihood ratio.

a Predictive properties after exclusion of patients with prior antibiotics.

## Discussion

This prospective multicenter study shows that, in patients with a suspected infection and abnormal vital signs and no previous antibiotics presenting to the Emergency Department, the adjusted OR of shaking chills is approximately six for the presence of bacteremia. Vomiting, on the other hand, was not associated with bacteremia. However, the predictive properties of shaking chills are affected by the viral incidence and shaking chills may not be useful to detect bacteremia during the influenza season.

The presence of chills has long been considered a clinical sign of severe infection and bacteremia but few modern studies have in a systematic and prospective way investigated this relationship. The vast majority of previous studies have extracted the presence of chills from medical records, which introduces errors of omission [27]. Only a few studies have prospectively analyzed the relationship between chills and bacteremia [3, 4, 6–9, 28], and none of them have included all patients at the ED where a blood culture had been obtained. Several studies have tried to develop clinical prediction rules to calculate the risk of bacteremia in patients seeking health care with suspected infection [3, 4, 11–16, 18–23]. The proposed clinical prediction rules contain between 6 and 20 variables, including clinical parameters, laboratory results, and anamnestic data. The results vary, but as Eliakim-Raz *et al*., showed in 2015, none of the developed tools are in clinical use [29]. The reasons for this may be because the scoring systems are difficult and time-consuming, require a specific diagnosis, and include variables that are not available in the emergency room.

Some studies include chills as a variable and show that 14–95% of patients with bacteremia and 9–75% of patients without bacteremia have chills [3, 4, 6, 11, 13, 15, 22, 30, 31]. The reason for this very large variation may be that the data usually derives from retrospectively reviewing clinical admission notes. Since the patient does not always specify the presence of chills and the clinician does not always record it, this information is not reliable. Another reason for the large variation may be that the study populations differ from each other.

In the present study, the positive predictive value for shaking chills was 32%, which is higher than several more complex models. However, this comes at the expense of a lower negative predictive value (85%) [6, 20, 21, 32]. For example, Shapiro *et al*. [21] included 13 independent predictors, including chills and vomiting, in a multivariate analysis, to create a clinical decision rule for when to order blood cultures at the ER. The rule had a PPV of 11% and an NPV of 99% for a positive blood culture. The advantage of this rule is that it can reduce the use of blood cultures in patients with a low likelihood of having bacteremia. However, it is complicated to use and partially relies on blood tests, many of which are not readily available in the emergency room when the decision to initiate antibiotic treatment is made.

The results of our study show that the absence of shaking chills does not exclude the risk of bacteremia. However, the presence of shaking chills indicates an elevated risk of having bacteremia, and therefore it should strongly encourage the clinician to order blood cultures. In our study, the presence of vomiting did not add any predictive capacity to the risk analysis, neither alone nor in combination with shaking chills.

An interesting finding was the unexpectedly high frequency of shaking chills among patients with viral infections. Among those with a probable or verified viral infection, 24% experienced shaking chills and among those with bacteremia (and no prior antibiotics) 38% experienced shaking chills. The high frequency of shaking chills among patients with viral infections evidently diminishes the predictive value of shaking chills in diagnosing bacteremia during periods when the likelihood for viral infections is high (e.g. during influenza season). A model was constructed to account for the incidence of viral infections. According to this model shaking chills had an OR of 31 (95% CI 4.60–280.82) when no viral infections were present whereas e.g. with a viral incidence of 17% the OR was 1. The high OR for shaking chills in this model is due to the fact that when viral disease is adjusted for then shaking chills is strongly associated with bacteremia. For example, when excluding all patients with viral infections, 11 out of 25 patients presenting with chills had bacteremia.

Furthermore, the impact of prior antibiotics was assessed. Patients that had received antibiotics prior to the ED encounter had a higher risk of having bacteremia than those that had not received prior antibiotics (OR 3.29, 95% CI 1.01–10.11). This is most likely explained by the fact that patients with prior antibiotics are a subgroup, which *per se* have a higher risk of bacteremia. Patients in the analyzed cohort were both infected as well as uninfected but among those with prior antibiotics a higher proportion had an infection and hence a higher risk for bacteremia. However, once adjusted for prior antibiotics the presence of chills does not seem to prognosticate bacteremia in patients with prior antibiotics whereas in patients without prior antibiotics chills are associated with bacteremia (OR 5.89, 95% CI 2.05–17.17).

The most important strength of our study is that the information about shaking chills and vomiting was carefully investigated by the including physicians at the Emergency Department upon initial patient assessment, as a predefined study variable. In comparison, in most other studies the information was extracted from retrospectively reviewing clinical admission notes, which is an uncertain source of anamnestic information. Furthermore, the patients were enrolled in the study regardless of their reason to visit the ED, which makes it a unique study population, since most other studies on chills are made only on patients presenting with fever. In this way, not only patients with suspected infection are included. Additionally, this is a large multicenter-study, including patients from three different sites and countries, why the results may be generalizable to many settings.

This study also has several limitations. Firstly, since only patients with at least one abnormal vital sign were included in the study, the results may not be generalized to patients with normal vital signs. Secondly, since the decision to order blood cultures were made by the attending clinician, only the patients where the clinician decided to order blood cultures qualified for inclusion in the analyses of bacteremia. Thus, there is an inclusion bias, where patients who already were suspected to have a high risk of infection were preferentially included into the cohort. However, of the 282 patients where blood cultures were not obtained, only 11 patients (4%) had experienced shaking chills compared to 34 patients (17%) among those 197 patients where a blood culture was obtained (*P* = 2 × 10^−6^). Thirdly, the study was conducted in non-malaria endemic countries and hence none of the included patients had malaria. In malaria-endemic countries the association between chills and bacteremia is most likely affected by the incidence of malaria. Fourthly, all patients included in the original study protocol were not asked about the presence of shaking chills, and therefore had to be excluded from this study, making it a possible inclusion bias. Fifthly, the study population is small, which means the conclusions have to be interpreted with caution. This is especially true for mortality; among the 197 patients included only four died during the first 72 h. Hence, we have not analyzed the association between chills and mortality.

Choosing which patients who should have blood cultures obtained and which patients who should have antibiotics administered is a delicate, but very common question at the emergency departments. A thorough inquiry about the clinical sign of shaking chills should be a natural part of the patient assessment. In conclusion, this study shows that shaking chills in patients presenting with abnormal vital signs at the emergency department is a good predictor of bacteremia. The tendency is higher when the incidence of viral infections is low or when a viral infection can be excluded. The presence of shaking chills under such circumstances represents a strong indication for obtaining blood cultures and empirical antibiotic therapy. The study also shows that vomiting was not associated with bacteremia. However, the findings could be considered preliminary given the small sample size of this study and further larger studies are warranted.

## Supplementary material

For supplementary material accompanying this paper visit https://doi.org/10.1017/S0950268820000746. click here to view supplementary material
