# Supplementary material for: Risk of bacteremia in patients presenting with shaking chills and vomiting - a prospective cohort study
Source: Epidemiol Infect. 2020 Mar 31;148:e86. doi: 10.1017/S0950268820000746 (PMC7189349; doi:10.1017/S0950268820000746)
Supplement: Supplementary file 1 [file S0950268820000746sup001.docx]

**Epidemiology and Infection**

**Title:** Risk of bacteremia in patients presenting with shaking chills and vomiting - a prospective cohort study

**Authors:** M. Holmqvist, M. Inghammar, L. I. Påhlman, J. Boyd, P. Åkesson, A. Linder, F. Kahn

**Supplementary material**

**Supplementary Table S1)** Baseline characteristics

|  | N | No chills  N=163 | Chills  N=34 | p-value |
| --- | --- | --- | --- | --- |
| **Site** | 197 |  |  | 0.5 |
| Lund |  | 62% (101) | 53% (18) |  |
| Bern |  | 21% (34) | 24% (8) |  |
| Vancouver |  | 17% (28) | 24% (8) |  |
| **Type of problem** | 197 |  |  | 0.02 |
| Verified bacterial infection |  | 33 % (53) | 41% (14) |  |
| Probable bacterial infection |  | 20% (33) | 21% (7) |  |
| Verified viral infection |  | 13% (21) | 18% (6) |  |
| Probable viral infection |  | 1% (1) | 9% (3) |  |
| Probable no infection |  | 17% (28) | 6% (2) |  |
| No infection |  | 17% (27) | 6% (2) |  |
| **Positive blood culture**  **Prior antibiotics** | 197  196 | 13% (21)  15% (24) | 32% (11)  21% (7) | 0.009  0.4 |
| **Vomiting** | 196 | 17% (28) | 30% (10) | 0.09 |
| **Fever** | 195 | 38% (61) | 68% (23) | 0.002 |
| **Age** | 197 | 71.3 (59.3-81.8) | 67.3 (53.6-79.2) | 0.3 |
| **Females** | 197 | 43% (70) | 44% (15) | 1 |
| **Comorbidities** |  |  |  |  |
| Cardiovascular | 197 | 54% (88) | 41% (14) | 0.2 |
| Respiratory | 197 | 27% (44) | 21% (7) | 0.5 |
| Diabetes | 197 | 23% (37) | 24% (8) | 1 |
| Renal | 197 | 20% (32) | 21% (7) | 1 |
| Malignancy | 197 | 20% (32) | 18% (6) | 1 |
| COPD | 197 | 13% (21) | 12% (4) | 1 |
| Liver | 197 | 4% (7) | 12% (4) | 0.1 |
| Immunodeficiency | 197 | 4% (7) | 6% (2) | 0.7 |
| No comorbidities | 197 | 18% (29) | 32% (11) | 0.06 |
| **Outcome** |  |  |  |  |
| ICU-admittance within 72 hours | 196 | 11% (18) | 6% (2) | 0.5 |
| Mortality within 72 hours | 197 | 2% (3) | 3% (1) | 0.5 |
| Number of dysfunctional organs | 197 |  |  | 0.5 |
| 0 |  | 25% (40) | 24% (8) |  |
| 1 |  | 32% (52) | 44% (15) |  |
| 2 |  | 26% (42) | 15% (5) |  |
| 3 |  | 6% (10) | 12% (4) |  |
| 4 |  | 6% (10) | 3% (1) |  |
| 5 |  | 3% (5) | 0% (0) |  |
| 6 |  | 1% (1) | 0% (0) |  |
| 7 |  | 2% (3) | 3% (1) |  |
| **Diagnoses** | 197 |  |  | 0.4 |
| Infections |  | 67% (110) | 88% (30) |  |
| Respiratory |  | 26% (42) | 21% (7) |  |
| Influenza |  | 9% (14) | 15% (5) |  |
| Genitourinary |  | 7% (12) | 15% (5) |  |
| Unspecified sepsis |  | 6% (10) | 6% (2) |  |
| Gastrointestinal |  | 5% (8) | 6% (2) |  |
| Other bacterial infection |  | 5% (8) | 3% (1) |  |
| Other viral infection |  | 4% (7) | 9% (3) |  |
| COPD exacerbation |  | 2% (4) | 3% (1) |  |
| Skin/soft tissue |  | 2% (3) | 6% (2) |  |
| Endocarditis |  | 1% (2) | 6% (2) |  |
| Central nervous |  | 0% (0) | 0% (0) |  |
| Non-infectious causes |  | 33% (53) | 12% (4) |  |
| Other |  | 7% (11) | 3% (1) |  |
| Gastro |  | 6% (9) | 0% (0) |  |
| Lung |  | 6% (9) | 3% (1) |  |
| Unspecified heart |  | 5% (8) | 0% (0) |  |
| Lung embolic |  | 3% (5) | 0% (0) |  |
| CNS |  | 2% (3) | 0% (0) |  |
| Orthopedic |  | 2% (3) | 0% (0) |  |
| Cerebrovascular |  | 1% (1) | 3% (1) |  |
| Intoxication |  | 1% (1) | 0% (0) |  |
| Diabetes |  | 1% (1) | 0% (0) |  |
| Heart rhythm |  | 1% (1) | 0% (0) |  |
| Vascular |  | 1% (1) | 0% (0) |  |
| Kidney |  | 0% (0) | 3% (1) |  |
| Acute myocardial infarction |  | 0% (0) | 0%(0) |  |
| Head trauma |  | 0% (0) | 0% (0) |  |
| Liver |  | 0% (0) | 0% (0) |  |
| Psychiatric |  | 0% (0) | 0% (0) |  |
| Seizures |  | 0% (0) | 0% (0) |  |

**Baseline characteristics of the cohort where blood cultures were obtained (n=197). Continuous variables are displayed with median and inter-quartile range. Categorical variables are displayed with proportions and numbers within brackets. Non-categorical variables are tested with Mann-Whitney U test and categorical variables with Fisher’s exact test.**
